# Tazarotene-Induced Gene 1 (TIG1) Interacts with Serine Protease Inhibitor Kazal-Type 2 (SPINK2) to Inhibit Cellular Invasion of Testicular Carcinoma Cells

**DOI:** 10.1155/2019/6171065

**Published:** 2019-11-25

**Authors:** Rong-Yaun Shyu, Chun-Hua Wang, Chang-Chieh Wu, Lu-Kai Wang, Mao-Liang Chen, Chan-Yen Kuo, Ming-Cheng Lee, Yi-Ying Lin, Fu-Ming Tsai

**Affiliations:** ^1^Department of Internal Medicine, Taipei Tzuchi Hospital, The Buddhist Tzuchi Medical Foundation, New Taipei City 231, Taiwan; ^2^Department of Dermatology, Taipei Tzuchi Hospital, The Buddhist Tzuchi Medical Foundation, New Taipei City 231, Taiwan; ^3^School of Medicine, Tzu Chi University, Hualien 970, Taiwan; ^4^Department of Surgery, Tri-Service General Hospital Keelung Branch, National Defense Medical Center, Keelung 202, Taiwan; ^5^Radiation Biology Core Laboratory, Institute for Radiological Research, Chang Gung University, Chang Gung Memorial Hospital, Linkou, Taoyuan 333, Taiwan; ^6^Department of Research, Taipei Tzuchi Hospital, The Buddhist Tzuchi Medical Foundation, New Taipei City 231, Taiwan

## Abstract

Tazarotene-induced gene 1 (TIG1) encodes a protein that is a retinoid-regulated tumor suppressor. TIG1 is expressed in most normal tissues, and downregulation of TIG1 expression in multiple cancers is caused by promoter hypermethylation. Kazal-type serine protease inhibitor-2 (SPINK2) is a serine protease inhibitor, and the SPINK protein family has been shown to inhibit the expression of urokinase-type plasminogen activator (uPA). In addition, increased levels of uPA and the uPA receptor were observed in testicular cancer tissues. This study demonstrated that TIG1 interacts with SPINK2 in NT2/D1 testicular carcinoma cells. TIG1 and SPINK2 were highly expressed in normal testis tissues, while low expression levels of TIG1 and SPINK2 were found in testicular cancer tissues. TIG1 inhibited cell invasion, migration, and epithelial–mesenchymal transition (EMT) of NT2/D1 cells. SPINK2 enhanced TIG1-regulated uPA activity and EMT suppression, while silencing SPINK2 alleviated TIG1-mediated EMT regulation, cell migration, and invasion. Therefore, the results suggest that the interaction between TIG1 and SPINK2 plays an important role in the inhibition of testicular cancer cell EMT, and suppression is mediated through downregulation of the uPA/uPAR signaling pathway.

## 1. Introduction

Tazarotene-induced gene 1 (TIG1), also known as retinoic acid receptor responder 1 (RARRES1), is a retinoic acid regulated tumor suppressor gene [1]. Downregulation of TIG1 in multiple cancers is mediated by common CpG hypermethylation in the TIG1 promoter region [2–7]. TIG1 belongs to the latexin family of putative cytoplasmic carboxypeptidase inhibitors, and it has been shown to regulate the *α*-tubulin tyrosination cycle via the ATP/GTP binding protein-like 2 (AGBL2) protein [8].

In addition to regulation of *α*-tubulin, which is related to mitochondrial function, ectopic TIG1 exhibits cell growth suppression and induction of autophagy in cervical, colon, and nasopharyngeal cancer cells [9–13]. Microarray analysis of mifepristone-inducible TIG1 expression in colon cells revealed G protein-coupled receptor kinase 5 (GRK5)-mediated TIG1-induced cell growth suppression through the Wnt and cAMP signaling pathways [9, 10]. The heat shock protein DNAJC8 and the transmembrane protein 192 have been shown to participate in TIG1-mediated glycolysis metabolism and autophagy regulation [11, 12]. Knockdown of TIG1 leads to enhanced invasion capacity of HK1-EBV nasopharyngeal cells [13]; however, the underlying mechanism of cell invasion mediated by TIG1 is still unclear.

The serine protease inhibitor Kazal-type protein family contains a Kazal domain located at the C-terminus, which is an evolutionary conserved protein domain of the serine protease inhibitor. SPINK1 has been considered a tumor-associated trypsin inhibitor, and overexpression of SPINK1 is a predictor of unfavorable outcomes in ovarian [14], prostate [15], liver [16], breast [17], and colon [18] cancers. In contrast, SPINK6 has been found to inhibit tumorigenesis in human hepatocellular carcinoma via the regulation of ERK1/2 activation [19]. SPINK-induced activities, which contribute to tumor growth, could be related to its target proteins, such as Kallikrein-related peptidase (KLK). KLK-mediated tumor growth is believed to modulate plasmin formation through the urokinase plasminogen-type activator (uPA) system [20]. Loss of SPINK7 has been shown to increase uPA activity in esophageal epithelial cells [21], indicating that SPINK7 may represent a key checkpoint in regulating mucosal barrier function in esophageal cells. Similarly, a recent study has shown that downregulation of SPINK13 promotes metastasis through the uPA system in ovarian cancer cells [22].

Numerous studies have demonstrated the activity of carboxypeptidase inhibitors in TIG1-mediated regulation of inflammation and mitochondrial function [8, 23]. We also identified TIG1 as a regulator of glucose metabolism via DNAJC8 and PKM2 proteins [11]. However, the mechanism by which TIG1 is involved in migration, invasion, or metastasis remains unknown. We used a yeast two-hybrid screen to demonstrate that TIG1 interacts with SPINK2. Because the SPINK protein family has been shown to inhibit uPA activity, which may contribute to epithelial-to-mesenchymal transition (EMT) and tumor metastasis, we hypothesized that SPINK2 might participate in TIG1-mediated inhibition of cancer cell invasion. Therefore, the current study investigated whether TIG1 affects the activity of uPA, which is negatively regulated by SPINK2, potentially preventing EMT and suppressing cell migration and invasion.

## 2. Materials and Methods

### 2.1. Quantitative Reverse Transcription PCR (RT-QPCR)

Human total RNA was purchased from Takara Bio USA, Inc. (San Jose, CA). Testis RNA samples were derived from four normal testis cells, three testicular embryonal carcinomas, and three testicular seminomas from Origene Technologies (Rockville, MD). Then, 1.5 *μ*g of RNA was first reverse transcribed into cDNA, and quantitative PCR was performed. Briefly, reverse transcription reactions were conducted in 20 *μ*L reaction mixtures containing 50 mM Tris-HCl (pH 8.3), 75 mM KCl, 3 mM MgC_l2_, 10 mM DTT, 2 *μ*M oligo (dT)_12–18_, 0.5 mM dNTP, and 10 units of M-MLV reverse transcriptase (Invitrogen, Carlsbad, CA). The cDNA mixture was incubated at 37°C for 1 h, and the enzyme was subsequently inactivated by heating at 72°C for 15 min. Quantitative PCR was then performed in triplicate in a 20 *μ*L reaction mixture containing 50 ng cDNA, 10 *μ*L Fast SYBR Green Master Mix (Applied Biosystems, Foster City, CA), and 100 nM of TIG1- or SPINK2-specific primers. PCR cycling conditions were as follows: a denaturing incubation at 95°C for 1 min followed by 40 cycles of denaturing at 95°C for 15 s and annealing and extension at 60°C for 1 min. These amplifications were performed in a thermal cycler (7900HT Fast Real-Time PCR System, Applied Biosystems). PCR primers used for amplification included the following: TIG1 (5′-CGTGGTCTTCAGCACAGAGCG-3′ and 5′-CCCAAACGTCCCTCACCTTCC-3′), SPINK2 (5′-TACCTTCGCAGCCTCTCTGA-3′ and 5′-AGCAGGGTCCATTTCGAATGAT-3′), and actin (5′-TCCCTGGAGAAGAGCTACG-3′ and 5′-GTAGTTTCGTGGATGCCACA-3′). Expression levels of TIG1 and SPINK2 were normalized to the mean of actin expression level.

### 2.2. Expression Vectors and SiRNAs

The pTIG1-myc-his expression vector was previously described [9]. To generate the pSPINK2-flag expression vector, SPINK2 cDNA was first amplified from human NT2/D1 testicular cancer cells using SPINK2-specific primers (5′-TGGCTAGCATGGCGCTGTCGGTGCTGCGC-3′ and 5′-GACTCGAGGCAGGGTCCATTTCGAATGATTTTA-3′). The amplified SPINK2 cDNA was then digested with *Nhe* I and *Xho* I followed bysubcloning into the *Nhe* I-*Xho* I sites of the PCR3.1-Flag vector. All SPINK2 siRNAs targeted against nucleotides 391–409 (5′-GAATGTACTCTGTGCATGA-3′), nucleotides 496–514 (5′-CACCTTCACTGGCAGACTA-3′), and nucleotides 508–526 (5′-CAGACTAGATAAATTGCAT-3′) were based on the GenBank accession NM_021114.3 and were synthesized by Sigma (Saint Louis, MO).

### 2.3. Cell Culture and Transfection

NT2/D1 testicular carcinoma cells were purchased from Bioresource Collection and Research Center (Hsinchu, Taiwan). NT2/D1 cells were cultured in Dulbecco's Modified Essential Medium (DMEM) containing 2 mM L-glutamine, 100 units/mL penicillin and streptomycin, and 10% fetal bovine serum (FBS) at 37°C in 5% CO_2_. For transfection, cells were first cultured in 24-well or 6-well plates at a density of 2 × 10^4^ or 1 × 10^5^ cells per well overnight. Plasmids and X-tremeGENE HP DNA Transfection Reagent (Sigma) were diluted in DMEM without serum at room temperature for 10–15 min. The X-tremeGENE HP DNA Transfection Reagent and plasmid complexes were then added to cells without removing the culture medium. Cell lysates were prepared 24 h after transfections were performed. Alternatively, cells were cultured in serum-free DMEM for an additional 12 h after cells were transfected for 24 h. Cells were subsequently harvested for cell migration and invasion assays.

### 2.4. Cell Viability Assay

NT2/D1 cells were cultured in 24-well plates overnight. Cells were then transfected with 250 ng pTIG1-myc-his expression vector along with 250 ng empty control vector or pSPINK2-flag expression vector for 24 h. The cells were cultured in DMEM without serum for 12 h followed by 24 h incubation in medium containing 1% FBS. Cells were incubated in the presence of the WST-1 reagent (Roche Diagnostics, Mannheim, Germany) for an additional 4 h. Culture medium was collected, and the absorbance (450–650 nm) of each sample was determined with a multifunctional microplate reader (Infinite F200, Tecan, Durham, NC, USA).

### 2.5. Cell Migration and Invasion Assays

NT2/D1 cells were seeded into 6-well plates overnight. Cells were then transfected with 1 *μ*g of expression vectors along with 30 *μ*M siRNAs for 24 h. After incubation for 24 h followed by serum-free starvation for 12 h, cells were collected and reseeded in DMEM without serum at a density of 2 × 10^4^ cells per well in the upper transwell insert with an 8 *μ*m pore size (Falcon, Becton Dickinson). In the cell invasion assay, 5 *μ*g/*μ*L matrix-gel (Becton Dickinson, Franklin Lakes, New Jersey) was coated onto a transwell insert, followed by cell seeding in the polycarbonate membrane insert. DMEM with 20% FBS was used as a chemoattractant in the lower well in both the cell invasion and migration assays. After 24 h of incubation, cells were fixed with methanol for 10 min at room temperature and were immediately placed at −20°C for overnight incubation. Cells were then stained with a 50 *μ*g/mL solution of propidium iodide (Sigma) for 30 min. After cells were washed twice with PBS, the number of cells on each transwell membrane was examined with a Nikon ECLIPSE 80i microscope (Nikon Instruments Inc., Melville, NY).

### 2.6. Immunoprecipitation and Western Blotting

Cells were plated in 10 cm dishes at a density of 2 × 10^6^ cells per dish overnight. After 24 h of transfection with 3 *μ*g TIG1-myc-his and 3 *μ*g SPINK2-flag expression vectors, cell lysates were prepared in modified RIPA buffer (20 mM Tris-HCl [pH 7.5], 100 mM NaCl, 1% NP−40, 30 mM sodium pyrophosphate) containing protease and phosphatase inhibitors. Cell lysate (500 *μ*g) proteins were incubated with 1 *μ*g of an anti-c-MYC (Sigma) or an anti-FLAG (Sigma) antibody at 4°C for 2 h, followed by the addition of 20 *μ*L of protein G plus/protein A-agarose (Calbiochem, Cambridge, MA). Samples were then incubated at 4°C for an additional 2 h. Alternatively, cell lysate (5 mg) proteins were first incubated with 2 *μ*g of an anti-TIG1 antibody (Santa Cruz Biotechnology, Santa Cruz, CA) or an anti-SPINK2 antibody (GeneTex Inc., Irvine, CA) and were then incubated with protein G plus/protein A-agarose at 4°C for 2 h. Samples were then washed with PBS three times, and immunoprecipitated complexes were then analyzed by western blotting. For western blotting, immunoprecipitated complexes or 15–50 *μ*g of cell lysate protein was resuspended in Laemmli buffer and then run on 12–15% SDS-PAGE gels. Proteins were separated by SDS-PAGE followed by electrotransfer to polyvinylidene fluoride membranes. Membranes were incubated in blocking buffer (5% nonfat dry milk, 0.1% Tween-20 in 1x PBS) at room temperature for 1 h followed by incubation in blocking buffer with anti-MYC, anti-FLAG, anti-TIG1, anti-SPINK2, anti-E-cadherin (Santa Cruz Biotechnology), anti-vimentin (Santa Cruz Biotechnology), or anti-actin (Sigma) antibody at 4°C for 12 h. After washing with 1x PBS containing 0.1% Tween-20 three times, membranes were incubated with horseradish peroxidase-conjugated goat anti-mouse or anti-rabbit antibodies at room temperature for 1 h. After washing, target protein bands were visualized by chemiluminescence (ECL detection kit, Amersham Biosciences, Bucks, UK), and images were subsequently analyzed with a ChemiDoc™ XRS+ System (Bio-Rad Laboratories, Hercules, CA).

### 2.7. Immunofluorescence Staining

Cells were seeded onto polylysine-coated coverslips in 6-well plates at 37°C overnight. After 24 h of transfection with 500 ng of TIG1-myc-his expression vector and 500 ng of SPINK2-flag expression vector, cells were washed with 1x PBS and were fixed with 4% paraformaldehyde in 1x PBS. Cells were then permeabilized with 0.1% Triton X-100 for 5 min on ice followed by blocking with 1x PBS containing 2% BSA at room temperature for 30 min. Cells were incubated in 1x PBS containing 2% BSA with either the anti-c-MYC or anti-Flag antibody at 4°C for 12 h followed by incubation in 1x PBS containing 2% BSA with the secondary antibodies Alexa Fluor 633 anti-mouse IgG and Alexa Fluor 488 anti-rabbit IgG (Invitrogen) at room temperature for 2 h. After washing three times with 1x PBS, cells were stained with DAPI and analyzed for TIG1-MYC and SPINK2-FLAG expression with a confocal microscope (Leica TCS SP5 scanner, Bensheim, Germany).

### 2.8. Measurement of uPA Activity

Cells were seeded in triplicate into 6-well plates in complete medium at 37°C overnight. After 24 h of transfection with the expression vectors or siRNAs, cells were incubated in serum-free DMEM for 24 h. Cell lysates were harvested, and uPA activity was determined using a SensoLyte AFC uPA activity assay kit (AnaSpec, San Francisco, CA).

### 2.9. Statistical Analysis

Results are expressed as mean ± SDs of at least three replicates. Statistical significance was determined by a one-way ANOVA, and a *p*-value <0.05 indicated a statistically significant difference.

## 3. Results

### 3.1. Downregulated TIG1 and SPINK2 Expression in Testicular Carcinoma Cells

**Figure 1:**
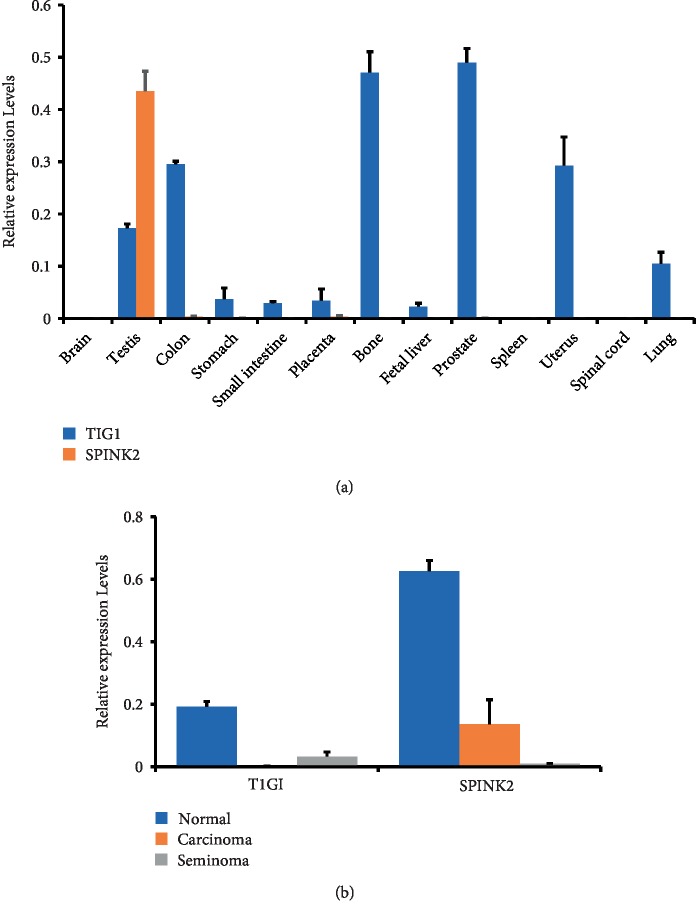
TIG1 and SPINK2 are highly expressed in testis tissues. Amplified cDNA samples prepared from total RNA of 13 different normal human tissues (a) or from total RNA of 4 normal, 3 carcinoma, or 3 seminoma tissues (b). Expression levels of TIG1 and SPINK2 were analyzed by quantitative real-time PCR. After normalizing expression to *β*-actin, relative expression levels are shown.

To explore the possible role of TIG1 and SPINK2 in regulating cell function, the distribution of TIG1 and SPINK2 in thirteen different normal tissues was first analyzed. TIG1 was highly expressed in testis, bone, uterus, and lung tissues, while SPINK2 mRNA was only detected in testicular tissues (Figure 1(a)). Since TIG1 and SPINK2 are highly expressed in testis tissue, we next detected their mRNA expression in normal testicular cells, testicular embryonal carcinomas, and testicular seminomas (Figure 1(b)). Decreased gene expression in TIG1 and SPINK2 was observed in cancer tissues, indicating that TIG1 and SPINK2 might participate in cancer development in testicular tissue.

### 3.2. TIG1 and SPINK2 Inhibit Cell Migration and Invasion in NT2/D1 Testicular Carcinoma Cells

**Figure 2:**
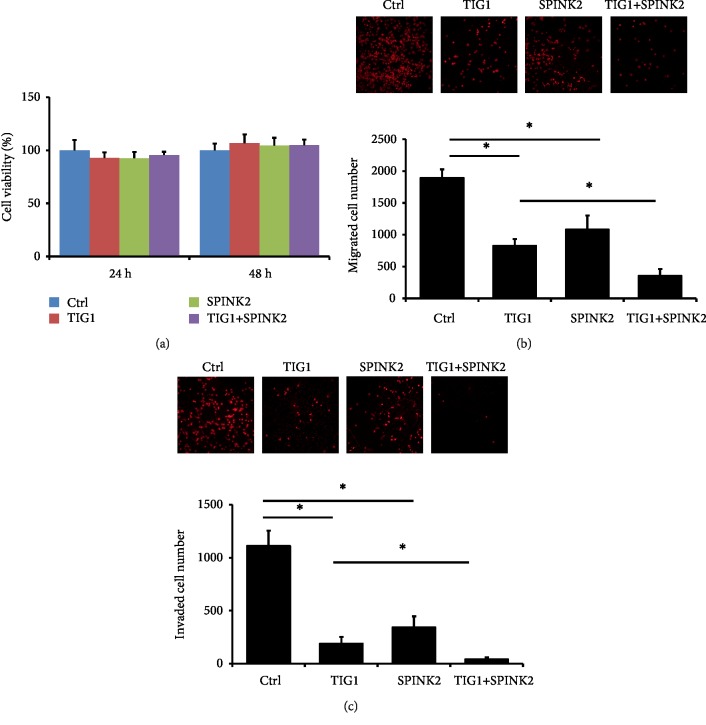
TIG1 and SPINK2 suppress cell migration and invasion. NT2/D1 cells were plated in triplicate in 24-well plates. After cells were transfected with TIG1-myc-his and SPINK2-flag expression vectors for 24 h, cells were serum starved for 12 h and were then incubated in medium containing 1% FBS for 24 h. Cell viability was determined by a WST-1 assay (a). NT2/D1 cells were transfected with the indicated expression vectors for 24 h. After serum starvation for 12 h, cells were added in triplicate into the upper transwell insert. Culture medium containing 20% FBS was used as a chemoattractant in the lower wells. Migratory cells (b), and invading cells (c) were stained with PI after 24 h incubation. Representative results from three independent experiments are shown, and the data are presented as the mean ± SD.^∗^*p* < 0.05.

Because of the decreased expression of TIG1 and SPINK2 in testicular carcinoma tissue, the effects of TIG1 and SPINK2 on cell viability in NT2/D1 cells were further determined. NT2/D1 cells were transfected with TIG1-myc-his or SPINK2-flag expression vectors for 24–48 h, and cell viability was examined using the WST-1 reagent. We detected no significant difference in cell viability in NT2/D1 cells expressing either TIG1-MYC or SPINK2-FLAG (Figure 2(a)). In addition, no cell death was observed, as determined by LDH release assays, in NT2/D1 cells transfected with TIG1 or SPINK2 expression vectors for 24–48 h (data not shown). In contrast, TIG1 or SPINK2 expression in NT2/D1 cells significantly inhibited cell migration by 56.4% and 42.9%, respectively, compared to cell migration of NT2/D1 cells transfected with empty control vector. Furthermore, the number of migrated cells decreased by 57.4% in TIG1and SPINK2 coexpressing cells compared to cells transfected with TIG1-myc expression vector for 24 h (Figure 2(b) and Supplementary [Supplementary-material supplementary-material-1]). Additionally, cell invasion decreased by 82.9% and 69.2% in NT2/D1 cells transfected with either the TIG1 or SPINK2 expression vector, respectively. Coexpressing TIG1 and SPINK2 in NT2/D1 cells further suppressed cell invasion by 78% compared to cell invasion of TIG1-expressing cells (Figure 2(c) and Supplementary [Supplementary-material supplementary-material-1]).

### 3.3. TIG1 Associates with SPINK2

**Figure 3:**
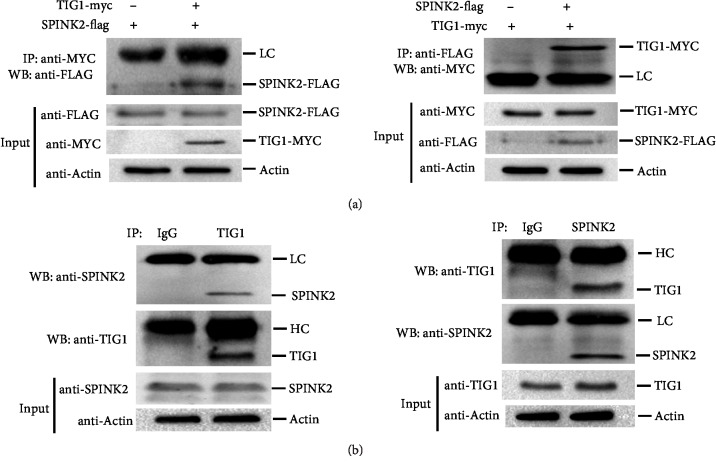
TIG1 associates with SPINK2. Cell lysates were prepared from NT2/D1 cells transfected with TIG1-myc-his and SPINK2-flag expression vectors for 24 h. The interaction between TIG1-MYC and SPINK2-FLAG was analyzed by coimmunoprecipitation and western blot analysis using anti-MYC or anti-FLAG antibodies (a). Cell lysates were prepared from NT2/D1 cells, and the interaction between TIG1 and SPINK2 was analyzed by coimmunoprecipitation using anti-TIG1 or anti-SPINK2 antibodies followed by western blot analysis (b).

**Figure 4:**
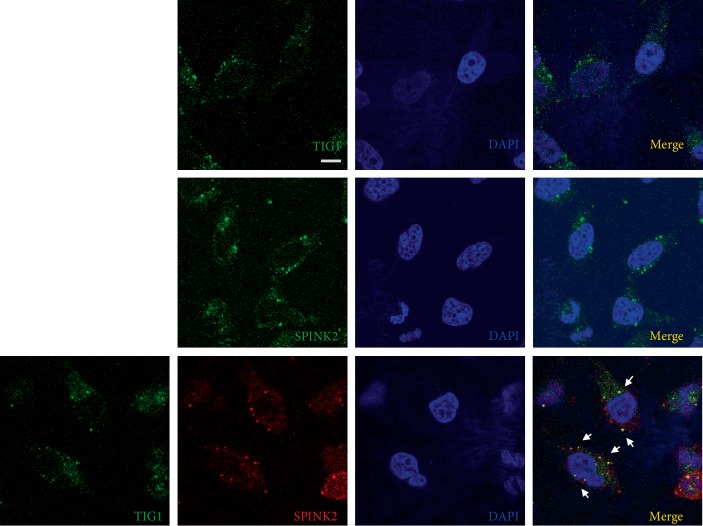
TIG1 co-localizes with SPINK2. NT2/D1 cells were cotransfected with TIG1-myc-his and SPINK2-flag expression vectors for 24 h. After fixation with 4% paraformaldehyde and permeabilization, cells were incubated with anti-MYC and anti-FLAG antibodies followed by incubation with an Alexa Fluor 488-conjugated goat anti-rabbit IgG or an Alexa Fluor 633-conjugated goat anti-mouse IgG antibody. After washing with 1x PBS, cells were stained with DAPI and were then analyzed for TIG1-MYC and SPINK2-FLAG expression with a confocal microscope. Scale bar: 10 *μ*m.

Interaction of TIG1 and SPINK2 was examined in a yeast two-hybrid screen. To confirm the interaction between TIG1 and SPINK2 within cells, coimmunoprecipitation was performed. TIG1-MYC was pulled down using anti-MYC antibody from the lysates of NT2/D1 cells cotransfected with TIG1-myc-his and SPINK2-flag expression vectors for 24 h. Coimmunoprecipitation results revealed that SPINK2-FLAG was present in the TIG1-MYC immunoprecipitated complexes (Figure 3(a)). Similarly, TIG1-MYC was incorporated into the SPINK2-FLAG complexes, as determined by a pull-down assay using an anti-FLAG antibody (Figure 3(a)). In addition to overexpression of TIG1 and SPINK2, we also examined the interaction between endogenous TIG1 and SPINK2 using TIG1- or SPINK2-specific antibodies. Coimmunoprecipitation results confirmed that endogenous TIG1 associates with SPINK2 (Figure 3(b)). We further verified sublocalization of TIG1 and SPINK2 within cells. Immunofluorescence staining images revealed that both TIG1 and SPINK2 exhibited punctate distribution at perinuclear organelles, and most TIG1 and SPINK2 proteins were colocalized (yellow) in cotransfected NT2/D1 cells (Figure 4).

### 3.4. TIG1 Suppresses EMT

**Figure 5:**
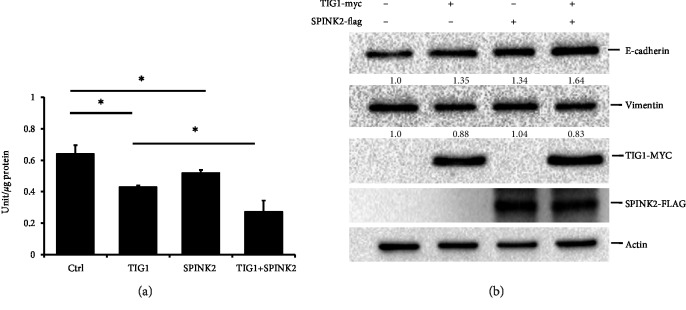
TIG1 and SPINK2 inhibit EMT. NT2/D1 cells were transfected with TIG1-myc-his and SPINK2-flag expression vectors for 24 h and were serum starved for 24 h. Cell lysates were prepared, and uPA activity was measured by enzyme immunoassay. Representative results from three independent experiments are shown, and data are presented as the mean ± SD. ∗*p* < 0.05 (a). Cell lysates from NT2/D1 cells transfected with TIG1-myc-his and SPINK2-flag expression vectors were prepared. Expression levels of E-cadherin, vimentin, TIG1-MYC, and SPINK2-FLAG were determined by western blot analysis.

The SPINK2 homolog has been shown to inhibit cell metastasis by suppressing levels of uPA protein in ovarian cells [22]; we next determined whether expression of TIG1 or SPINK2 in NT2/D1 cells affects the activity of uPA. Expressing TIG1 and SPINK2 in NT2/D1 cells led to downregulation of uPA activity by 33% and 18.9%, respectively. Coexpressing SPINK2 further promoted TIG1-mediated suppression of uPA activity by 63.7% (Figure 5(a) and Supplementary [Supplementary-material supplementary-material-1]). Having found that the uPA system is involved in EMT processes, we next examined the effects of TIG1 and SPINK2 on expression of E-cadherin and vimentin. Expression of TIG1 and SPINK2 resulted in increased E-cadherin protein levels by 1.35- and 1.34-fold, respectively, while levels of vimentin were decreased by 12.3% in TIG1-expressing cells (Figure 5(b)). Levels of E-cadherin were enhanced by 20.9% in NT2/D1 cells coexpressing TIG1 and SPINK2 compared to levels in TIG1-expressing cells (Figure 5(b)).

### 3.5. SPINK2 Silencing Alleviates TIG1-Mediated EMT Suppression

**Figure 6:**
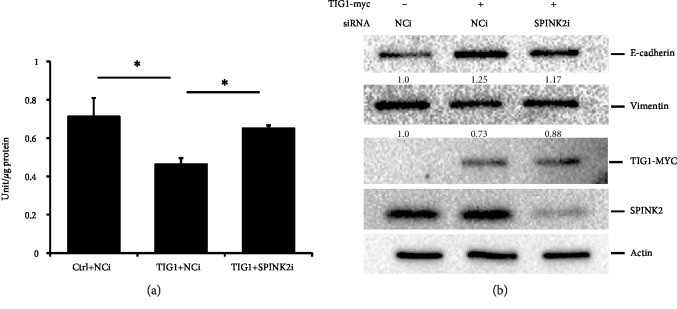
SPINK2 siRNAs alleviate TIG1-regulated EMT. NT2/D1 cells were transfected with TIG1-myc-his expression vector along with control (NCi) or SPINK2 siRNAs for 24 h and were serum starved for 24 h. Cell lysates were prepared, and uPA activity was measured by enzyme immunoassay. Representative results from three independent experiments are shown, and data are presented as the mean ± SD. ^∗^*p* < 0.05 (a). Cell lysates from NT2/D1 cells transfected with TIG1-myc-his expression vector along with NCi or SPINK2 siRNAs were prepared. Expression levels of E-cadherin, vimentin, TIG1-MYC, and SPINK2 were determined by western blot analysis.

**Figure 7:**
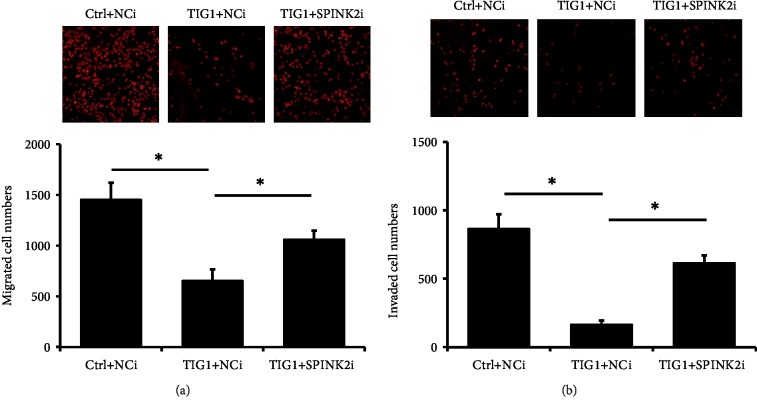
SPINK2 siRNAs alleviate TIG-regulated cell migration and invasion. NT2/D1 cells were transfected with TIG1-myc-his expression vectors along with NCi or SPINK2 siRNAs for 24 h and were serum starved for 12 h. Migratory cells (a) and invading cells (b) were stained with PI after 24 h incubation. Representative results from three independent experiments are shown, and data are presented as mean ± SD. ∗*p* < 0.05.

Since SPINK2 enhanced TIG1-mediated E-cadherin expression, we next examined whether knockdown of SPINK2 would affect TIG1-mediated EMT processes. uPA activity was decreased by 34.9% in TIG1-expressing NT2/D1 cells, and silencing of SPINK2 significantly alleviated TIG1-mediated uPA activity suppression by 73.7% (Figure 6(a)). Furthermore, enhanced levels of E-cadherin by TIG1 were decreased by 63.5% in TIG1-expressing cells cotransfected with SPINK2 siRNA (Figure 6(b)). Decreased levels of vimentin in response to TIG1 expression were also restored by 55.1% in TIG1- and SPINK2-siRNA coexpressing cells (Figure 6(b) and Supplementary [Supplementary-material supplementary-material-1]). In addition, silencing expression of SPINK2 significantly alleviated TIG1-mediated cell migration and invasion suppression by 51.1% (Figure 7(a) and Supplementary [Supplementary-material supplementary-material-1]) and 64.2% (Figure 7(b) and Supplementary [Supplementary-material supplementary-material-1]), respectively.

## 4. Discussion

Our results indicate that expression of TIG1 in NT2/D1 testicular carcinoma cells leads to decreased uPA activity, EMT reduction, and inhibition of cell migration and invasion. We also found that TIG1 interacts with SPINK2 within cells, and coexpression of SPINK2 enhanced TIG1-mediated inhibition of uPA activity and cell invasion, while TIG1-mediated cell invasion was reversed in SPINK2-silenced cells. These observations suggest that TIG1 regulates testis cell invasion and migration and may influence the uPA system via interaction with the SPINK2 protein.

A previous study showed that TIG1 induces expression of GRK5, subsequently decreasing cell proliferation and increasing cell death in colorectal carcinoma cells [9]. However, TIG1-induced decreases in cell viability were not observed in TIG1-expressing NT2/D1 testicular carcinoma cells. Furthermore, no apparent changes in cell morphology were observed when TIG1 was expressed in NT2/D1 cells (Figure 4). The different fates that occur in distinct subtypes of cancer cells expressing TIG1 might be caused by TIG1-targeting proteins. Moreover, regulation of the uPA system by TIG1 in testis cells may not occur in other cancer cells because SPINK2 is expressed in testis tissue only (Figure 1).

In mammalian cells, serine proteases can be broadly classified into secreted proteases and membrane-anchored proteases. Secreted serine proteases, such as uPA, have been shown to regulate a range of biological events, including tissue repair, the immune system, and nutrient uptake. In contrast, membrane-anchored proteases, such as GPI-anchored uPAR, have roles in various physiological events, including the epithelial barrier, embryonic development, and tissue differentiation [24]. Dysregulated expression of the uPA/uPAR system results in cell invasion in multiple cancer cell lines [25–31]. In contrast, expression levels of uPA and uPAR have been shown to be increased in testicular germ cell tumors compared to levels in adjacent normal tissues [32]. This suggests that the uPA/uPAR system has an important role in cancer growth and tumorigenesis in testis tissue.

SPINK1 was first as a member of the Kazal domain-containing protein family [33]. The primary function of SPINK1 is to serve as a pancreatic and intestinal serine protease inhibitor and to balance levels of trypsinogen and trypsin. However, SPINK1 overexpression, which was observed in different types of cancer [34], may be partially due to SPINK1 having structural homology with the epidermal growth factor [18, 35, 36]. In contrast to SPINK1, there are few reports in the literature concerning the role of SPINK2 on normal physiological function or tumorigenesis. The tissue-specific distribution of SPINK2 may be the cause. Although the role of SPINK2 in the testis is still not clear, SPINK2 might regulate cell migration and invasion in testicular cancer cells through the uPA/uPAR system; this is supported firstly by the similar activity of homologous proteins [21, 22]. Second, our current study observed an interaction between TIG1 and SPINK2, and the association of these two proteins was involved in suppressing the activity of uPA and subsequent effects on EMT and cell migration in testis cancer cells.

Our study demonstrated that expression of TIG1 inhibits cell migration and invasion in testicular carcinoma cells. SPINK2 can interact with TIG1 and enhance TIG1-mediated EMT suppression. Silencing of SPINK2 alleviated the effects induced by TIG1 in testicular carcinoma cells. These results suggest that TIG1 and SPINK2 can be developed as novel molecular targets to prevent metastasis of testicular carcinoma.

## Data Availability

All data used to support the findings of this study are included within the article.

## Supplementary Materials

Supplementary Materials Supplementary Table 1. TIG1 and SPINK2 suppress NT2/D1 cell migration and invasion. Supplementary Table 2. Effect of TIG1 and SPINK2 on uPA activity in NT2/D1 cells. Supplementary Table 3. Effect of SPINK2 siRNA on TIG1-regulated uPA activity in NT2/D1 cells. Supplementary Table 4. SPINK2 siRNA alleviates TIG-regulated cell migration and invasion.
