# Supplementary material for: Tazarotene-Induced Gene 1 (TIG1) Interacts with Serine Protease Inhibitor Kazal-Type 2 (SPINK2) to Inhibit Cellular Invasion of Testicular Carcinoma Cells
Source: Biomed Res Int. 2019 Nov 25;2019:6171065. doi: 10.1155/2019/6171065 (PMC6899300; doi:10.1155/2019/6171065)
Supplement: Supplementary Materials — Supplementary Table 1. TIG1 and SPINK2 suppress NT2/D1 cell migration and invasion. Supplementary Table 2. Effect of TIG1 and SPINK2 on uPA activity in NT2/D1 cells. Supplementary Table 3. Effect of SPINK2 siRNA on TIG1-regulated uPA activity in NT2/D1 cells. Supplementary Table 4. SPINK2 siRNA alleviates TIG-regulated cell migration and invasion. [file 6171065.f1.pdf]

Supplementary Table 1. TIG1 and SPINK2 suppress NT2/D1 cell migration and invasion

		Cell number	*p*-value
Migrated cells	Ctrl	1892 ±134.5	
	TIG1	824.7 ± 106.5	1.64877E-05[a]
	SPINK2	1080.7 ±220.9	0.00015[a]
	TIG1+SPINK2	351.3 ±110.5	0.00019[b]
Invaded cells	Ctrl	1110.3 ± 143.3	
	TIG1	189.3 ± 62.7	0.00116[a]
	SPINK2	341.7 ± 104.5	0.00025 [a]
	TIG1+SPINK2	41.7 ± 18.6	0.01724[b]

[a] $p < 0.05$ cells transfected with TIG1 or SPINK2 plasmid compared with cells transfected with empty vector (Ctrl group). [b] $p < 0.05$ cells cotransfected with TIG1 and SPINK2 plasmids compared with cells transfected with TIG1 plasmid.

Supplementary Table 2. Effect of TIG1 and SPINK2 on uPA activity in NT2/D1 cells

	uPA activity (unit/μg protein)	*p*-value
Ctrl	0.64 ± 0.06	
TIG1	0.43 ± 0.01	0.00063[a]
SPINK2	0.52 ± 0.02	0.00028[a]
TIG1+SPINK2	0.27 ± 0.07	0.00284[b]

[a] $p < 0.05$ cells transfected with TIG1 or SPINK2 plasmid compared with cells transfected with empty vector (Ctrl group). [b] $p < 0.05$ cells cotransfected with TIG1 and SPINK2 plasmids compared with cells transfected with TIG1 plasmid.

Supplementary Table 3. Effect of SPINK2 siRNA on TIG1-regulated uPA activity in NT2/D1 cells.

	uPA activity (unit/μg protein)	*p*-value
Ctrl + NCi	0.71 ± 0.09	
TIG1 + NCi	0.46 ± 0.03	0.00096[a]
TIG1+ SPINK2i	0.65 ± 0.02	1.99263E-05[b]

[a] p <0.05 cells transfected with TIG1 plasmid and control (NCi) siRNA compared with cells transfected with empty vector and NCi. [b] p <0.05 cells cotransfected with TIG1 plasmid and SPINK2 siRNA compared with cells transfected with TIG1 plasmid and NCi.

Supplementary Table 4. SPINK2 siRNA alleviates TIG-regulated cell migration and invasion.

		Cell number	*p*-value
Migrated cells	Ctrl + NCi	1450.7 ±169.2	
	TIG1 + NCi	651 ± 114	0.00014[a]
	TIG1 + SPINK2i	1060 ±88.4	5.24801E-05[b]
Invaded cells	Ctrl + NCi	1110.3 ± 143.3	
	TIG1 + NCi	189.3 ± 62.7	0.00225[a]
	TIG1 + SPINK2i	341.7 ± 104.5	0.00026[b]

[a] p <0.05 cells transfected with TIG1 plasmid and NCi compared with cells transfected with empty vector and NCi. [b] p <0.05 cells cotransfected with TIG1 plasmid and SPINK2 siRNA compared with cells transfected with TIG1 plasmid and NCi.
